# Integration of HIV Care with Primary Health Care Services: Effect on Patient Satisfaction and Stigma in Rural Kenya

**DOI:** 10.1155/2013/485715

**Published:** 2013-05-07

**Authors:** Thomas A. Odeny, Jeremy Penner, Jayne Lewis-Kulzer, Hannah H. Leslie, Starley B. Shade, Walter Adero, Jackson Kioko, Craig R. Cohen, Elizabeth A. Bukusi

**Affiliations:** ^1^Family AIDS Care and Education Services, Kisumu, Kenya; ^2^Kenya Medical Research Institute, Nairobi, Kenya; ^3^Department of Epidemiology, University of Washington, Seattle, USA; ^4^Department of Family Practice, University of British Columbia, Vancouver, Canada; ^5^Departments of Obstetrics, Gynecology and Reproductive Sciences, University of California San Francisco, San Francisco, CA, USA; ^6^Center for AIDS Prevention Studies, University of California San Francisco, San Francisco, CA, USA; ^7^Ministry of Public Health and Sanitation, Government of Kenya, Kisumu, Kenya

## Abstract

HIV departments within Kenyan health facilities are usually better staffed and equipped than departments offering non-HIV services. Integration of HIV services into primary care may address this issue of skewed resource allocation. Between 2008 and 2010, we piloted a system of integrating HIV services into primary care in rural Kenya. Before integration, we conducted a survey among returning adults ≥18-year old attending the HIV clinic. We then integrated HIV and primary care services. Three and twelve months after integration, we administered the same questionnaires to a sample of returning adults attending the integrated clinic. Changes in patient responses were assessed using truncated linear regression and logistic regression. At 12 months after integration, respondents were more likely to be satisfied with reception services (adjusted odds ratio, aOR 2.71, 95% CI 1.32–5.56), HIV education (aOR 3.28, 95% CI 1.92–6.83), and wait time (aOR 1.97 95% CI 1.03–3.76). Men's comfort with receiving care at an integrated clinic did not change (aOR = 0.46 95% CI 0.06–3.86). Women were more likely to express discomfort after integration (aOR 3.37 95% CI 1.33–8.52). Integration of HIV services into primary care services was associated with significant increases in patient satisfaction in certain domains, with no negative effect on satisfaction.

## 1. Introduction

Funding targeted for HIV care programs in sub-Saharan Africa has produced tremendous results over the past several years, most notably the delivery of antiretroviral therapy to almost 4 million people in sub-Saharan Africa by 2009 [1, 2]. In Kenya, the number of people receiving antiretroviral therapy has increased from about 11,000 in 2003 to more than 138,000 patients in 2007 largely as a result of receiving the President's Emergency Plan for AIDS Relief (PEPFAR) funds [3, 4]. This kind of directed “vertical” funding (for specific disease areas instead of for general improvements in primary health care) has allowed for specialized staff training, more rapid and efficient program implementation, and better-equipped facilities—including free laboratory services and medications—as HIV programs have been scaled-up [5]. These results may not have been possible in such a short time using an integrated approach to health care delivery. 

Nevertheless, the HIV epidemic has increased pressure on the Kenyan health care system as a whole by increasing the workload for health personnel—whose numbers have not increased proportionally to the demand [6]—and straining infrastructure capacity and public expenditure. Vertical HIV programs may exacerbate the situation further, as the concentration of resources leads to HIV clinics being better equipped and their workers better compensated with financial and nonmonetary incentives [7, 8]. The resulting attrition of personnel from general health services may weaken important primary health care services [8, 9]. 

Integration of HIV services into primary care can be defined in multiple ways. In the context of this paper, we define it as colocation and sharing of services and resources for HIV care and primary care, such as clinic space, clinicians, health education, pharmacy, laboratory services, and training. Integration of HIV services into primary care addresses the issue of skewed resource allocation, allowing people to access the health care they require regardless of HIV status. Available evidence suggests that integration offers several potential advantages at this point in the response to HIV in sub-Saharan Africa [10, 11]. Colocation of HIV and primary care services maximizes the use of available health facility structures and ensures that funds targeted for rehabilitation or construction of HIV care facilities will also benefit primary care [7]. Decentralization of HIV care services into primary health care clinics results in greater acceptability of services, increased referrals and enrolments into HIV care, and improved patient retention compared to services at specialized hospitals [12–15]. Patient outcomes may also be better, and costs lower, at primary health clinics [16, 17]. Support systems such as monitoring and evaluation (M&E), supply-chain management, laboratory networking, and counseling services can also be shared when HIV services are integrated into primary care services. Pfeiffer et al. reported that integration results in the inclusion of antiretroviral therapy (ART-) related data into national health databases [7]. Such joint information systems also increase efficiency [18]. In addition, integration allows staff to share the workload for HIV-positive and HIV-negative patients, resulting in more efficient use of resources and reduced patient waiting time [18]. A program of decentralization and integration of HIV care services into the primary care system in Mozambique reported improvements in access to care, quality of care, and efficiency in service delivery [7]. Integration may increase the positive effect of resources allocated to HIV: a study in Rwanda provides observational evidence that introduction of HIV care services results in improved staff capacity at primary health care facilities due to the additional in-service training given to health care workers [19]. Increases in the uptake of other services, especially antenatal care, were also associated with integration, suggesting that a mutually beneficial interaction can be created between HIV care and other primary care services. 

Despite the evidence suggesting system-wide and national-level benefits to integration, the patient- and service-level effects are unclear. Whereas the overall benefits of integration are likely to trickle down to patients and providers, it is also possible that the resulting reorganization of health care delivery may disrupt service provision and potentially cause dissatisfaction among patients, particularly in the short term. Further, integration of specialized services into primary care services may not always result in better patient- and service- level outcomes [20]. For example, integration of HIV services with sexual and reproductive health services may be hindered by increased patient burden, inadequate staffing, and resistance from existing health care workers [21]. Similarly, integrating services for sexually transmitted infections into routine health services may result in lower utilization and reduced patient satisfaction [22]. Also, integrating services for family planning and maternal and child health care into routine primary care may reduce knowledge of family planning [23]. 

There is minimal evidence to suggest that stigma associated with HIV care may reduce with integration [24]. Stigma may be reduced if a patient's HIV status cannot be determined by the general public simply by observing the physical location where a patient is receiving care. However, integration of services may lead to other opportunities for accidental disclosure of one's HIV status, so the effect of integration on stigma is unclear. A study assessing HIV/AIDS stigma on health service utilization demonstrated that anticipated HIV stigma can be a barrier to HIV testing acceptance among pregnant women at clinics that were part of a cluster-randomized trial evaluating the effect of integration of HIV and antenatal care services [25].

In 2008, a system that integrated HIV services into primary care was piloted in rural health facilities in Nyanza Province in western Kenya. We assessed the patient-level effect of this integration with a focus on patient satisfaction and perceived stigma.

## 2. Methods

From December 2008 to January 2010, we conducted an evaluation of the pilot integration project at three health facilities to assess the patient-level effect of integration. Family AIDS care and education services (FACES) implemented the integration in collaboration with the Suba District Health Management Team of the Kenya Ministry of Health. FACES is a collaboration between the Kenya Medical Research Institute (KEMRI) and the University of California, San Francisco (UCSF), funded through the US Centers for Disease Control (CDC)/PEPFAR [26]. 

### 2.1. Study Site

The pilot was conducted in Suba District in Western Kenya, which has a population of 214,463 [27] spread over the mainland and 10 inhabited islands. The 26.3% HIV prevalence in Suba is the highest in Kenya, which has a national average of 7.1% [3, 28]. At the time of the pilot, there were 40 health facilities of varying capacity staffed by about 200 healthcare workers, 144 of whom were nurses. In contrast, the Suba District Annual Operations Plan 2008-2009 recommends 934 healthcare workers, including 591 nurses [6]. Of the 40 facilities, 20 provided HIV care and treatment services to a total of approximately 15,000 patients. Three health facilities underwent integration and were evaluated during the pilot period: one subdistrict hospital and two health centers, representing the two main levels of health care in the district. Although the subdistrict hospital and health centers chosen for this evaluation represent different levels of health care, both types of health facilities served similar patient populations (rural), had similar patient volumes in HIV care (approximately 400 patients booked each month), had similar catchment populations (subdistrict hospital, 7,500; health centers, 7,200), had similar levels of staffing cadres providing clinical services (one clinical officer supported by nurses), and had the same availability of space to allow colocation of services.

### 2.2. Features of Integration

We addressed the different aspects of integration as shown in Table 1. The implementation of these activities also constitutes our definition of integration of HIV care with primary health care services. Further features of the integrated health facility are described below. 

#### 2.2.1. Clinic Space and Clinicians

Before integration, HIV-positive patients were seen separately on one to four dedicated days each week. After integration, they were seen on any day of the week in the general outpatient clinic; all patients followed a uniform patient flow, and patient load was evenly spread out among the existing staff. 

#### 2.2.2. Health Education

Integrated health education was conducted for all patients regardless of HIV status in a single shared waiting bay. This approach was seen as a way of reducing stigma against HIV and broadening health education reach.

#### 2.2.3. Pharmacy/Supplies

With integration, all drugs were stored in the same storage space and dispensed from the same pharmacy; supply and inventory management at the clinics under evaluation were also integrated. The integrated pharmacy became the central repository and dispensing area for therapeutic food supplements (for eligible HIV-infected patients and HIV-negative children of certain ages) that would otherwise have to be stored in a separate food store. Orders for all drugs and food supplements were made within the same supply-chain network. Training and mentorship for pharmacy and supplies management were offered to both Ministry of Health (MOH) and FACES staff who then worked together in the integrated pharmacy and were able to share the daily workload.

#### 2.2.4. Laboratory

Introduction of HIV services brought with it a well-equipped central laboratory at the district hospital; after integration, these lab facilities were used to conduct other tests not necessarily related to HIV. For example, a new automated biochemistry machine to be used for measuring liver enzymes to monitor the progress of patients on ART was also used for other patients when clinically indicated. Supportive supervision for the district lab network prior to integration was infrequently conducted by the MOH District Medical Laboratory Technician; however, after integration it was conducted by a combined FACES and MOH team. Laboratory supervision was more frequent and regular after integration because FACES had a more reliable transport system.

#### 2.2.5. Clinical Mentorship and Training

Before integration, only providers working in the HIV clinic underwent mentorship by experienced providers from FACES during weekly visits. After integration, all providers underwent mentorship. All clinical officers and nurses were offered training for HIV-related and non-HIV-related topics such as Rational Use of ART in Adults, Comprehensive Pediatric HIV Care, Integrated Management of Adult and Adolescent Illness, Prevention of Mother to Child Transmission of HIV, and Psychosocial Counseling for Pediatrics.

### 2.3. Study Participants

Study eligibility (inclusion criteria) at baseline included adult patients ≥18-year old who were HIV-positive and already enrolled in HIV care (including patients pre-ART and those on ART), attending a return visit to study facilities in rural Suba District. Exclusion criteria included patients <18-year old, patients not enrolled in HIV care, and patients enrolling in care that day. Inclusion and exclusion criteria remained the same at the followup with one exception: individuals enrolled in HIV care as well as those accessing general outpatient services were included, reflecting the integration of these services.

Patient participation involved a self-administered questionnaire on service satisfaction and perceived stigma. The questionnaire was available in the language of the patient's choice (Dholuo, Kiswahili, or English), and all data collection was anonymous. A sample size of 10% of the patients enrolled into HIV care at each health facility was targeted: 94 of the 941 (474 at the subdistrict hospital and 467 at the health centers) patients actively enrolled at baseline, 107 of the 1,065 (521 at the subdistrict hospital and 544 at the health centers) patients actively enrolled at three-month follow up, and 142 of the 1,421 (673 at the subdistrict hospital and 748 at the health centers) patients actively enrolled at 12-month followup, for an overall sample target of approximately 343 patients. A consecutive convenience sampling approach was used. Trained Monitoring and Evaluation (M&E) staff employed by FACES approached each departing patient throughout the day, determined eligibility verbally, obtained verbal consent, and carried out the assessment. Patients were advised that they were free to participate or not without affecting their services, and no incentives were provided. Willing participants completed the self-administered questionnaires in private and upon completion placed the questionnaires in a secure box to maintain privacy. The assessment was conducted over a 3-day period at baseline (December 2008) and followup (March 2009 and January 2010).

### 2.4. Measures

#### 2.4.1. Patient Satisfaction

Patient health facility satisfaction was assessed using a questionnaire that included questions adapted from the AIDS Clinical Research Trials Group (ACTG) tool. The questionnaire is a standard FACES program tool that was initially adapted, piloted, and validated for the local population at FACES-supported health facilities in Kisumu District and then used for this evaluation in Suba District (both in Nyanza Province, Kenya). Items included satisfaction with the office (courtesy and helpfulness), with clinicians (ability to listen, client respect, sufficient time with clients), and with each of seven departments: reception, community health assistants (who provide health talks and measure vital signs), health talks, nursing/counseling, laboratory, clinical, and pharmacy. Individual items addressed wait time, perceived usefulness of HIV education, and overall satisfaction with the clinic. Each item included three or four response options, such as “Very satisfied”, “Satisfied,” and “Not satisfied;” or “All the time,” “A lot of the time,” “Some of the time,” and “None of the time”. The two measures of office satisfaction and the three measures of clinician performance were each combined into linear scales for analysis. All other satisfaction measures were coded as binary responses with only the highest level of satisfaction coded as one.

#### 2.4.2. Perceived Stigma

Patient perception of stigma at the health facility was assessed using three items adapted from standardized items validated for use in this context [29]. Items covered privacy and confidentiality, equitable treatment for HIV-positive people, and discomfort receiving care at the health facility due to the possibility of other people finding out one's HIV status. Responses were grouped into agreement and disagreement for analysis.

#### 2.4.3. Data Management and Analysis

The data were entered and cleaned using SPSS Software (SPSS for Windows, Version 14.0 SPSS Inc., Chicago, IL, USA) and were merged across clinic sites and time before being analyzed using Stata 11.0 (StataCorp, College Station, TX, USA). Change from baseline was assessed using truncated linear regression (office satisfaction and clinician performance) and logistic regression (all other outcomes) with robust standard errors. All models were assessed for effect modification by sex and analyzed within sex if the interaction term with either time point was significant at *P* < 0.20. Survey language, sex, and being on ART were included as confounders in the final models based on showing an association with the outcome in unadjusted analysis (*P* < 0.20).

#### 2.4.4. Ethical Review

Ethical review committee approval was obtained locally and internationally; the protocol was reviewed for human subject concerns and approved by the KEMRI Ethical Review Committee and UCSF Committee on Human Research.

## 3. Results

Of the targeted sample of 343 surveys, 295 (86.0%) surveys were collected cumulatively over the three time points: 58 participants at baseline (61.7% of the 94 targeted), 104 participants at three months (97.3% of the 107 targeted), and 133 participants at 12 months (93.6% of the 142 targeted).  Table 2 shows patient demographics; of the 256 respondents whose sex is known, 94 (36.7%) were men and 162 (63.3%) were women. At baseline 28 (51.9%) were on ART, with this proportion increasing to 61 individuals (58.7%) at three months and 78 (60.9%) at 12-month followup. These consistently high percentages demonstrate that a large proportion of respondents were in HIV care even when the survey was administered to an integrated patient population. 

### 3.1. Patient Satisfaction

Patient satisfaction with the clinic office and with each department remained high throughout the assessment period (Table 3). By 12 months after integration, respondents had higher odds of being very satisfied with reception services (adjusted odds ratio, aOR 2.71, 95% CI 1.32–5.56), HIV education (aOR 3.28, 95% CI 1.92–6.83), and wait time (aOR 1.97, 95% CI 1.03–3.76) (Table 4). Women rated clinician performance more favorably at both the 3-month (*P* < 0.001) and 12-month assessment (*P* = 0.007) compared to baseline (Table 5). Respondent ratings of overall satisfaction declined significantly by the 3-month assessment (aOR of reporting very satisfied 0.45, 95% CI 0.21–0.95), although by the end of the assessment, this association was no longer present.

### 3.2. Perceived Stigma

All stigma-related items showed significant effect modification by sex (Table 5). At 12 months post-integration, men reported significantly higher levels of agreement that people with HIV were treated the same as others (aOR 17.81, 95% CI 1.83–173.12), while women's responses did not change significantly. Patient agreement with providers maintaining privacy and confidentiality did not change significantly within either sex during the assessment period. Men were less likely to agree that they were not comfortable receiving care at the clinic by the 3-month survey (aOR 0.05, 95% CI 0.00–0.48). Women were more likely to express discomfort at both time points compared to baseline: 3-month aOR 2.65, 95% CI 1.01–6.99; 12-month aOR 3.37, 95% 1.33–8.52. 

## 4. Discussion

Our findings suggest that integrating HIV care with primary care services does not negatively affect individual patients and may offer some benefits that extend beyond the health-system level to the individual patient. Assessment of the patient-level effect suggests that patient satisfaction remained high and that integration did not heighten perceived stigma. Before and after integration, patients generally agreed that care was provided confidentially and equitably regardless of HIV status although women expressed increased discomfort with receiving care at integrated clinics. In rural Kenya, other investigators from our group have found that overall client satisfaction with integrated HIV services among pregnant women is associated with satisfaction with administrative staff, satisfaction with health professionals, and convenient wait times and encounters with a receptionist [30].

As an initial assessment of the patient-level effect of integration, these findings may be a promising indication that integration may extend the resources concentrated within HIV care to a broader patient population without diminishing patients' perceived satisfaction. Patient education sessions appeared to have a greater effect when done in an integrated setting. The significant increase in satisfaction with HIV education as well as reception and wait times may be an indication that the increased staff training positively affected patient-provider interaction. The increased satisfaction with HIV education may have also resulted because the mixed population of HIV-positive and general patients found the sessions more beneficial than HIV-positive patients alone, or because of the broader scope of topics after integration. While we found that patients trusted healthcare providers to keep information confidential and to treat all patients equally to similar degrees before and after integration; women were less comfortable about receiving care at the integrated clinic. Since the follow-up samples included HIV-negative patients, it is possible that this finding relates to their discomfort with integrated care or that it represents continued high levels of apprehension regarding others suspecting one is HIV positive. The effect of integrated care on inadvertent disclosure bears further investigation. Overall, our findings suggest that despite the challenges involved in integrating HIV care into routine health care, it is possible to pursue integration without significant disruption of patients' experience, and in fact with some benefits observable even within a 12-month period. 

Our finding that patient satisfaction remained high with integration may be evidence that the restructuring required to achieve the system-wide benefits of integration [10, 11] does not result in patient dissatisfaction. Our findings that stigma did not significantly worsen with integration support some of the findings by Topp et al. [24].

### 4.1. Limitations

This study provides important integration findings. However, there are limitations to consider. The first limitation was the absence of a control group. It is possible that the changes observed could have been due to factors beyond the intervention. Second, the health facilities and the small number of patients interviewed within them may not be representative of the target population, limiting the generalizability of the findings. However, the proportion of respondents recruited out of the targeted number of participants increased substantially from 62% at baseline to greater than 90% at the followup. Third, the patient population changed between baseline and followup due to the nature of the intervention. In particular, the inability to distinguish between patients receiving HIV care and general care patients in the follow-up surveys limits the ability to determine how these two different patient groups responded to the intervention. Although we controlled for ARV status in multivariate analysis, we are unable to fully identify the impact of the intervention on patients receiving HIV care versus general patients. However, the consistently high percentages of patients on ART were an indication that a large proportion of respondents were in HIV care even when the survey was administered to an integrated patient population. By interviewing only patients who attended clinic and only tracking the number of willing participants, we were unable to capture the experiences of those who may have avoided clinic due to integration or who may have refused to participate, potentially introducing bias. Individual confounding factors may also influence the findings. Fourth, the questionnaire was self-administered; therefore, only literate respondents completed the questionnaire. In addition, the sampling strategy was not systematic and was therefore subject to selection bias from the research team. Other biases inherent in our evaluation design included social desirability bias and courtesy bias. Finally, the study may have benefitted from qualitative interviews to provide more insight into the results, especially in relation to improvements (or decline) in service delivery. However, this evaluation was done in the context of a mature HIV program in a real-world setting. We demonstrate the utility of operational research within HIV programs to improve program quality.

## 5. Conclusions

This study is a step towards developing a model of care that integrates HIV care into primary care in resource-limited settings. Given the potential positive aspects, it is worth exploring integration as one innovative way of improving primary care services that receive little donor funding, while at the same time maintaining the achievements of donor-supported HIV care at the patient level. Larger, cluster-randomized or stepped-wedge and longitudinal studies should be conducted to confirm these findings and address other critical issues, including the effect of integration on quality of care, long-term health outcomes, and cost-effectiveness.

## Figures and Tables

**Table 1:** Comparison of features of health facilities in an evaluation of HIV service integration in Suba District, Kenya.

	Patient support center (HIV clinic) before integration	Out-patient department (OPD) before integration	Integrated facility
Clinic space	Separate room for HIV clinical care	Separate room for primary care services other than HIV	All clinical rooms used for both HIV and other primary care services

Clinical staff	One clinician (clinical officer or nurse) dedicated to providing HIV care	Other clinicians attending to patients seeking primary care exclusive of HIV	All clinicians attend to all patients regardless of HIV status

Health education at waiting bay	General health education with emphasis on HIV care, treatment, and nutrition	General health education with equal emphasis on all topics. Messages about HIV mostly relate to prevention	General health education on all health topics including HIV prevention, care, and treatment

Pharmacy/Supplies	Staff extensively trained and mentored on commodity management and supply chain logistics especially relating to ART	Little or no training on commodity management. No mentorship. Poor supply chain hence frequent drug stock-outs	Intensive on-job training and mentorship on commodity management. Streamlined supply chain; less frequent drug stock-outs

Laboratory	Lab samples sent to well-stocked central lab at district hospital. Tests available for HIV-positive patients include CD4, CBC, hematology, microscopy, biochemistry, and immunology. More reliable supply of reagents; specimen transport network for remote sites	Labs poorly equipped. Tests available: Hb, pregnancy, and simple microscopy. Frequent stock out of reagents	Samples that cannot be processed locally sent to central lab at district hospital. Specimen transport network used for all specimens. Training and mentorship offered to all staff.

Focused HIV training and mentorship	Clinician well trained in HIV care (adult and pediatric ART, PMTCT); weekly mentorship by more experienced clinicians	Some staff trained but not actively practicing; no clinical mentorship	All staff trained in at least one HIV care course. Weekly mentorship and on-job training for all staff

**Table 2:** Patient demographics in an evaluation of HIV service integration in Suba district, Kenya (*n* = 295).

	Baseline	3-month followup	12-month followup	Total
	*N* (%)	*N* (%)	*N* (%)	*N* (%)
Male	18 (36.0%)	32 (35.2%)	44 (38.3%)	94 (36.7%)
Female	32 (64.0%)	59 (64.8)	71 (61.7%)	162 (63.3%)

On ART	28 (51.9%)	61 (58.7%)	78 (60.9%)	167 (58.4%)
Not on ART	26 (48.2%)	43 (41.4%)	50 (39.1%)	119 (41.6%)

**Table 3:** Patient satisfaction and stigma responses over time in an evaluation of HIV service integration in Suba district, Kenya.

	Baseline (n = 58)	3 months (n = 104)	12 months (n = 133)
	N (%)	N (%)	N (%)
Overall satisfaction			
Very satisfied	34 (59.65)	48 (46.60)	93 (74.40)
Satisfied	22 (38.60)	51 (49.51)	28 (22.40)
Not satisfied	1 (1.75)	4 (3.88)	4 (3.20)
Office courtesy			
All the time	35 (61.40)	68 (66.02)	101 (76.52)
A lot of the time	14 (24.56)	19 (18.45)	23 (17.42)
Some of the time	7 (12.28)	16 (15.53)	7 (5.30)
None of the time	1 (1.75)	0 (0.00)	1 (0.76)
Office helpfulness			
All the time	36 (66.67)	74 (71.84)	86 (65.65)
A lot of the time	11 (20.37%)	17 (16.50)	33 (25.19)
Some of the time	6 (11.11)	12 (11.65)	8 (6.11)
None of the time	1 (1.85)	0 (0.00)	4 (3.05)
Doctor listening			
All the time	30 (53.57)	68 (66.67)	87 (66.92)
A lot of the time	16 (28.57)	20 (19.61)	31 (23.85)
Some of the time	9 (16.07)	13 (12.75)	8 (6.15)
None of the time	1 (1.79)	1 (0.98)	4 (3.08)
Doctor shows respect			
All the time	34 (62.96)	69 (66.99)	91 (70.54)
A lot of the time	11 (20.37)	20 (19.42)	24 (18.60)
Some of the time	7 (12.96)	11 (10.68)	11 (8.53)
None of the time	2 (3.70)	3 (2.91)	3 (2.33)
Doctor time			
All the time	29 (53.70)	64 (62.14)	78 (59.54)
A lot of the time	17 (31.48)	28 (27.18)	31 (23.66)
Some of the time	6 (11.11)	9 (8.74)	15 (11.45)
None of the time	2 (3.70)	2 (1.94)	7 (5.34)
Wait time			
Just right	28 (49.12)	47 (46.53)	80 (61.07)
A bit long	26 (45.61)	45 (44.55)	34 (25.95)
Much too long	3 (5.26)	9 (8.91)	17 (12.98)
HIV education			
Very useful	32 (58.18)	63 (61.17)	105 (80.15)
Useful	11 (20.00)	38 (36.89)	22 (16.79)
Not at all useful	12 (21.82)	2 (1.94)	4 (3.05)
Reception			
Very satisfied	30 (54.55)	54 (51.92)	97 (74.05)
Satisfied	25 (45.45)	46 (44.23)	27 (20.61)
Not satisfied	0 (0.00)	4 (3.85)	7 (5.34)
Community health assistants			
Very satisfied	20 (34.48)	40 (38.46)	64 (48.12)
Satisfied	35 (60.34)	64 (61.54)	64 (48.12)
Not satisfied	3 (5.17	0 (0.00)	5 (3.76)
Nurses or counselors			
Very satisfied	26 (52.00)	50 (48.08)	86 (68.80)
Satisfied	20 (40.00)	53 (50.96)	34 (27.20)
Not satisfied	4 (8.00)	1 (0.96)	5 (4.00)
Lab			
Very satisfied	28 (56.00)	47 (45.19)	80 (62.99)
Satisfied	17 (34.00)	53 (50.96)	37 (29.13)
Not satisfied	5 (10.00)	4 (3.85)	10 (7.87)
Clinician			
Very satisfied	29 (56.86)	47 (45.19)	80 (62.50)
Satisfied	20 (39.22)	55 (52.88)	42 (32.81)
Not satisfied	2 (3.92)	2 (1.92)	6 (4.69)
Pharmacy			
Very satisfied	25 (50.00)	48 (46.15)	84 (65.12)
Satisfied	19 (38.00)	49 (47.12)	36 (27.91)
Not satisfied	6 (12.00)	7 (6.73)	9 (6.98)
Privacy & confidentiality			
Strongly agree	34 (60.71)	49 (48.04)	82 (63.57)
Agree	18 (32.14)	44 (43.14)	36 (27.91)
Disagree	4 (7.14)	9 (8.82)	6 (4.65)
Strongly disagree	0 (0.00)	0 (0.00)	5 (3.88)
People with HIV treated the same as others			
Strongly agree	28 (50.00)	45 (43.69)	81 (62.31)
Agree	17 (30.36)	40 (38.83)	36 (27.69)
Disagree	9 (16.07)	16 (15.53)	8 (6.15)
Strongly disagree	2 (3.57)	2 (1.94)	5 (3.85)
Not comfortable receiving care here			
Strongly agree	23 (42.59)	23 (22.33)	57 (46.72)
Agree	13 (24.07)	39 (37.86)	36 (29.51)
Disagree	13 (24.07)	21 (20.39)	14 (11.48)
Strongly disagree	5 (9.26)	20 (19.42)	15 (12.30)

**Table 4:** Regression analyses of changes in patient satisfaction over time in an evaluation of HIV service integration in Suba district, Kenya.

Outcome	3 months	12 months
	*β* (95% CI)	
Office courtesy and helpfulness^†^	0.40 (−0.32, 1.21)	0.61 (−0.08, 1.31)
	AOR (95% CI)	
Overall satisfaction	**0.45 **(**0.21**,** 0.95**)	1.32 (0.62, 2.78)
Satisfaction: group talks and vitals (CCHAs)^‡^	1.11 (0.53, 2.31)	1.55 (0.77, 3.13)
Satisfaction: reception^‡^	1.02 (0.50, 2.11)	**2.71 **(**1.32**,** 5.56**)
Satisfaction: nurses^‡^	0.74 (0.34, 1.61)	1.90 (0.89, 4.04)
Satisfaction: lab^‡^	0.75 (0.34, 1.67)	1.23 (0.57, 2.78)
Satisfaction: clinicians^‡^	0.59 (0.27, 1.26)	1.53 (0.73, 3.20)
HIV education^$^	1.29 (0.64, 2.62)	**3.28 **(**1.92**,** 6.83**)
Wait time^$^	1.01 (0.51, 1.98)	**1.97 **(**1.03**,** 3.76**)

^†^Controlling for being on ARVs, *n* = 286.

^$^Controlling for being on ARVs and language of survey, *n* = 280.

^‡^Controlling for being on ARVs, language of survey, sex.

**Table 5:** Regression analyses of changes in patient satisfaction and perceived stigma stratified by sex over time in an evaluation of HIV service integration in Suba District, Kenya.

Outcome	Stratum	3 months	12 months
		Coefficient, *β* (95% CI)	
Doctor respect, time, and listening^$^	Males (*n* = 91)	*β* = −1.22 (−3.38, 0.94)	*β* = 0.19 (−1.52, 1.91)
	Females (*n* = 157)	*β* = **2.83 **(**1.44**,** 4.21**)	*β* = **1.63** (**0.44**,** 2.83**)
		AOR (95% CI)	
Satisfaction: pharmacy	Males (*n* = 88)	0.82 (0.22, 3.11)	3.64 (0.94, 14.09)
	Females (*n* = 154)	0.62 (0.25, 1.56)	1.05 (0.42, 2.61)
People with HIV treated the same as others^$^	Males (*n* = 89)	0.71 (0.12, 4.26)	**17.81 **(**1.83**,** 173.12**)
	Females (*n* = 154)	1.82 (0.60, 5.51)	1.92 (0.58, 6.32)
Privacy & confidentiality^$^	Males (*n* = 88)	0.65 (0.06, 7.20)	5.98 (0.52, 68.70)
	Females (*n* = 153)	0.27 (0.02, 2.96)	0.41 (0.04, 3.87)
Not comfortable receiving care here^$^	Males (*n* = 88)	**0.05 **(**0.00**,** 0.48**)	0.46 (0.06, 3.36)
	Females (*n* = 149)	**2.65 **(**1.01**,** 6.99**)	**3.37 **(**1.33**,** 8.52**)

^†^Controlling for being on ARVs.

^$^Controlling for being on ARVs and language of survey.
